# Grafting of R_4_N^+^-Bearing Organosilane on Kaolinite, Montmorillonite, and Zeolite for Simultaneous Adsorption of Ammonium and Nitrate

**DOI:** 10.3390/ijerph191912562

**Published:** 2022-10-01

**Authors:** Wang Peng, Zhanpeng Cui, Hongyan Fu, Hongkai Cao, Ming Chen, Dachao Zhang, Wuhui Luo, Sili Ren

**Affiliations:** 1 Jiangxi Key Laboratory of Mining & Metallurgy Environmental Pollution Control, Jiangxi University of Science and Technology, Ganzhou 341000, China; 2 School of Resources and Environmental Engineering, Jiangxi University of Science and Technology, Ganzhou 341000, China; 3 Ganzhou Technology Innovation Center for Mine Ecology Remediation, Ganzhou 341000, China

**Keywords:** ammonium, nitrate, organosilane, aluminosilicate, grafting

## Abstract

Modification of aluminosilicate minerals using a R_4_N^+^-bearing organic modifier, through the formation of covalent bonds, is an applicable way to eliminate the modifier release and to maintain the ability to remove cationic pollutants. In this study, trimethyl [3-(trimethoxysilyl) propyl] ammonium chloride (TM) and/or dimethyl octadecyl [3-(trimethoxysilyl) propyl] ammonium chloride (DMO) were used to graft three aluminosilicate minerals, including calcined kaolinite (Kaol), montmorillonite (Mt), and zeolite (Zeol), and the obtained composites were deployed to assess their performance in regard to ammonium (NH_4_^+^) and nitrate (NO_3_^−^) adsorption. Grafting of TM and/or DMO had little influence on the crystal structures of Kaol and Zeol, but it increased the interlayer distance of Mt due to the intercalation. Compared to Kaol and Zeol, Mt had a substantially greater grafting concentration of organosilane. For Mt, the highest amount of loaded organosilane was observed when TM and DMO were used simultaneously, whereas for Kaol and Zeol, this occurred when only DMO was employed. ^29^Si-NMR spectra revealed that TM and/or DMO were covalently bonded on Mt. As opposed to NO_3_^−^, the amount of adsorbed NH_4_^+^ was reduced after TM and/or DMO grafting while having little effect on the adsorption rate. For the grafted Kaol and Zeol, the adsorption of NH_4_^+^ and NO_3_^−^ was non-interfering. This is different from the grafted Mt where NH_4_^+^ uptake was aided by the presence of NO_3_^−^. The higher concentration of DMO accounted for the larger NO_3_^−^ uptake, which was accompanied by improved affinity. The results provide a reference for grafting aluminosilicate minerals and designing efficient adsorbents for the co-adsorption of NH_4_^+^ and NO_3_^−^.

## 1. Introduction

Aluminosilicate minerals have been extensively modified by physical and/or chemical methods to further enhance their properties for use in environmental remediation and the synthesis of mineral–polymer nanocomposites [1,2]. One of the most common techniques is to use surfactants as the modifiers, particularly cationic alkyl quaternary ammonium salts [3]. In this instance, the cationic surfactants are initially anchored on the external and/or internal surface of the minerals via electrostatic attraction, and after the negative charges are neutralized, the counterion-accompanying surfactants (in molecular form) start to load in the solid phase via hydrophobic interaction [4]. Even though intensive washing procedures are typically applied to remove the loosely bound surfactants in order to minimize the leaching, the release of surfactants into the surrounding solution seems inevitable due to the weak interactions and the dissolubility of the counterion-accompanying surfactants [5,6]. Increasing the intensity of the electrostatic interactions by using other cationic modifiers with denser positively charged sites, such as gemini surfactants [7] and polymers [8], has been suggested as an effective solution to this issue and exhibits promising results. However, the desorption of cationic modifiers from the mineral surface may still occur in some hostile solutions with high ionic strength, aggravating the leaching [9]. Therefore, the grafting of modifiers in covalent bonding seems the only applicable way. Organosilane provides silanol to condensate with the hydroxyl groups on the mineral surfaces and also introduces some functional groups (e.g., –NH_2_ or –SH) to boost the adsorption of pollutants [10,11].

Using organosilane as the bridge for the surface modification has been well reviewed; the surface’s reactivity, the silane characteristics, and the reaction conditions were identified as the decisive factors for successful silylation [5]. One of the most researched organosilanes is 3-aminopropyltriethoxysilane (APTES). APTES-grafted clay minerals exhibit a favorable performance in the sequestration of heavy metal cations from aqueous solution through the formation of an inner-sphere complex [12]; however, they only work well for anions (e.g., CrO_4_^2−^, HAsO_4_^2−^) in acidic solutions where the protonation of –NH_2_ leads to the formation of the outer-sphere complex [12,13]. Introducing the quaternary ammonium (–R_4_N^+^) group could reduce the impact of solution pH on anion adsorption [14]. Numerous studies have demonstrated the remarkable affinity that R_4_N^+^-bearing surfactant functionalized aluminosilicates have for weakly hydrated anions (such as ClO_4_^−^, TcO_4_^−^, NO_3_^−^) [14,15,16]. Unmodified aluminosilicate minerals are capable of adsorbing cations through cation exchange because the framework of aluminosilicate minerals is typically negatively charged as a result of isomorphic substitutions (e.g., octahedrally coordinated Al^3+^ is populated by Mg^2+^) [17]. Therefore, adding R_4_N^+^ groups to aluminosilicate minerals may assure the simultaneous removal of cationic and anionic pollutants from the solution [9]. However, the routinely used alkyl quaternary ammonium salts neutralize the negatively charged adsorption sites of the minerals, making them less available for cationic pollutants [9]. Using organosilane with R_4_N^+^ groups to graft the aluminosilicate minerals may not result in the occupation of the negatively charged adsorption sites and may resolve such a paradox.

Recently, a typical R_4_N^+^-bearing organosilane, (3-trimethoxysilylpropyl) octadecyl dimethyl ammonium chloride, was applied to modify high-purity bentonite in order to distinguish the loading modes of cation exchange and covalent grafting; the content ratio of the modifier via cation exchange to that via the covalent bond was 84%:16% and 40%:60% for the original and acid-activated bentonite, respectively [18]. This suggests that loading R_4_N^+^ groups through electrostatic interaction seems inevitable. However, the stability test in hexane and 1.4 M NaCl solution demonstrated the negligible release of the R_4_N^+^-bearing organosilane, implying its promising application for pollutant adsorption. Thereafter, Dedzo et al. [19] used (3-trimethoxysilylpropyl) tetradecyl dimethyl ammonium chloride to modify a synthetic saponite for the identification of grafting sites and adsorption of the anionic Congo red. Unfortunately, the adsorption of cationic pollutant was not performed using the modified saponite. However, based on the proposed structure, it would be possible to simultaneously adsorb cationic and anionic pollutants. Notably, different from bentonite, intercalation was not observed for the original saponite. In other words, the aluminosilicate species is also an important factor that influences the modification using R_4_N^+^-bearing organosilane, which has been rarely reported.

**Figure 1:**
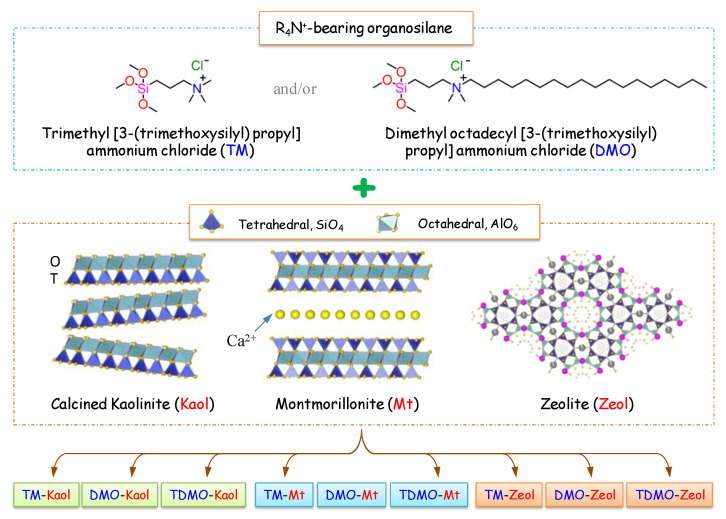
Grafting of the selected aluminosilicate minerals using two R_4_N^+^-bearing organosilanes.

Therefore, on one hand, three typical aluminosilicate minerals—calcined kaolinite (Kaol), montmorillonite (Mt), and zeolite (Zeol)—with significantly different physicochemical properties were applied in this study as substrates to better understand the grafting characteristics of R_4_N^+^-bearing organosilane (Figure 1). Notably, the calcined rather than the pristine kaolinite was used mainly because (1) the original kaolinite is non-swellable and may show similar grafting characteristics on the external surface as Zeol, (2) the calcined Kaol still possesses a considerable number of chemical reaction sites available for organosilane [20], and (3) the calcined Kaol has been rarely reported to graft by R_4_N^+^-bearing organosilane but is widely used as an additive for the synthesis of clay/polymer nanocomposites. On the other hand, even though most studies only focus on removing a single pollutant from the environment, the grafted minerals were sequentially used to test their performance in the simultaneous removal of ammonium (NH_4_^+^) and nitrate (NO_3_^−^) ions, given the common co-contamination of surface and/or ground water [21,22] and the environmental risks [22,23] of NH_4_^+^ and NO_3_^−^. Because the molecular structure of the organosilane may influence the grafting characteristics [24,25] and indirectly affect the decontamination performance [26,27], two R_4_N^+^-bearing organosilanes with different lengths of alkyl tails are selected for comparison (Figure 1).

## 2. Materials and Methods

### 2.1. Materials

Trimethyl [3-(trimethoxysilyl) propyl] ammonium chloride (TM, 50% in methanol) and dimethyl octadecyl [3-(trimethoxysilyl) propyl] ammonium chloride (DMO, 65% in methanol) were purchased from Macklin Biochemical Co., Ltd. (Shanghai, China). Kaol (Sinopharm Chemical Reagent Co., Ltd., Shanghai, China), Mt (Titan Scientific Co., Ltd., Shanghai, China), and Zeol (Sinopharm Chemical Reagent Co., Ltd., Shanghai, China) were used directly without further purification, and the compositions (Table 1) were determined by X-ray fluorescence spectrometer (Axios max, PANalytical, Almelo, Netherlands). Two adsorbates (i.e., NH_4_Cl and NaNO_3_) and other reagents used in the chromogenic reaction for spectrophotometry were of analytic grade. Ultrapure water was produced by a lab water system (Exceed-Ad-16, TangshiKangning Science Development Co., Ltd., Chengdu, China) and applied as the solvent for all synthesis and adsorption experiments.

### 2.2. Preparation of the Grafted Kaol, Mt, and Zeol

Two grams of Kaol, Mt, or Zeol were each dispersed in 200 mL of deionized water under vigorous stirring for 30 min. Different amounts of silane coupling agents, i.e., 5.6 mL of TM, 8.6 mL of DMO, or 2.3 mL TM + 4.3 mL DMO, were added drop wise into each dispersion. Sequentially, in order to perform the grafting reaction, the obtained mixture was transferred into a microwave and ultrasound combined system (XO-SM50, Atpio, Nanjing, China) operated under the following conditions: radiation for 60 min at 60 °C with operating powers of 500 and 450 W for the microwave and ultrasound, respectively. The final products were washed 3 times with ethanol/water (1:1 in volume, 50 mL) and pure water for the last round via the centrifuge (5000 rpm), lyophilized (18 h), and sieved (*d* < 149 µm). The resulting composites were named as organosilane-aluminosilicates, such as TM-Kaol, DMO-Mt, and TDMO-Zeol (Figure 1).

### 2.3. Solid Characterization

The X-ray reflection patterns of the composites were recorded using an Empyrean X-ray diffractometer (PANalytical, Almelo, The Netherlands) with Cu Kα radiation operated at 40 kV, 40 mA. Thermogravimetric analysis was performed on a thermal gravimetric analyzer (TG/DTA 6300, Seiko, Chiba, Japan) up to 900 °C at a heating rate of 10 °C/min with a N_2_ flow rate of 200 mL/min. Solid-state ^29^Si NMR spectra were collected on an Avance III HD spectrometer (Bruker, Germany) and the chemical shifts were standardized using tetramethylsilane (0 ppm).

### 2.4. Batch Adsorption Experiment

The prepared bulk aqueous solution with 1.0 g/L of NH_4_^+^-N or NO_3_^−^-N was used to acquire the adsorption systems with different concentrations of two adsorbates. In the study of the adsorption kinetics, the initial concentrations of NH_4_^+^-N and NO_3_^−^-N were set as 50 and 30 mg/L, respectively, and a solid-to-liquid ratio of 20 mg/25 mL was deployed. A batch of this mixture was shaken on a gyratory shaker (200 rpm) at 25 °C. At predetermined intervals, two parallel vials were retrieved and the mixture was filtered (cellulose acetate membrane with a pore size of 0.45 μm) to measure the residual concentrations of NH_4_^+^-N (*λ* = 420 nm) and NO_3_^−^-N (*λ* = 220 nm) using spectrophotometry. According to the mass balance, the amount of adsorbed NH_4_^+^-N or NO_3_^−^-N on the adsorbent was calculated and applied to depict the curves as a function of adsorption time to ensure the equilibrium. A cascade of adsorption solutions with different concentrations of NH_4_^+^-N ad NO_3_^−^-N were prepared to examine the influence of the initial concentration (Table S1). To get reliable results, the real concentrations of the adsorbates in the starting solution were also measured. With a solid-to-liquid ratio of 0.8 g/L, the equilibrium concentrations of two adsorbates in the solution after being shaken for 4 h, were determined to assess the adsorption performance. Each adsorption test was carried out in duplicate and the average value was presented.

## 3. Results and Discussion

### 3.1. Solid Characterization

#### 3.1.1. X-ray Diffraction

**Figure 2:**
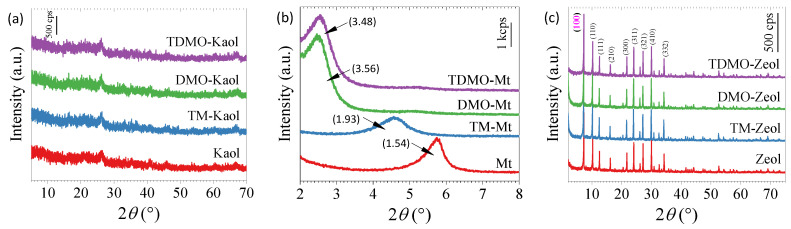
XRD patterns of the original and grafted (**a**) Kaol, (**b**) Mt, and (**c**) Zeol.

The chemical formulae of original kaolinite and Mt are normally expressed as Al_2_Si_2_O_5_(OH)_4_ and M*_z_*[(Si_4-*x*_, Al*_x_*)(Al_2-*y*_, Mg*_y_*)O_10_(OH)_2_]·*n*H_2_O (M refers to the mono- or divalent exchangeable cations, such as Na^+^ and Ca^2+^), respectively [28,29]. In contrast to the original kaolinite, Kaol underwent calcination and lost most of the hydroxyl groups, but it retained its reactivity when exposed to water [20]. As shown in Figure 2a, the characteristic 001 reflections of the original kaolinite at diffraction angles (2*θ*) close to 12° was not observed, and only a weak reflection at 2*θ* ≈ 27° assigned to the quartz was visible. The grafting of TM and/or DMO showed negligible influence on the crystalline structure of Kaol. Due to the swelling property of Mt, the XRD patterns for the original and grafted Mt at low 2*θ* were recorded (Figure 2b). The original Ca-Mt shows the 001 reflection at 2*θ* = 5.7°, corresponding to a 0.58-nm distance of interlayer space (*d*_001_—0.96 nm). The higher *d*_001_ values for the grafted Mt suggest that TM and/or DMO were successfully intercalated into the interlayer space. Due to the longer alkyl tail of DMO, DMO-Mt exhibits a significantly higher *d*_001_ value when compared to TM-Mt. The small difference in *d*_001_ values between DMO-Mt and TDMO-Mt further implies that the organosilane with the longer alkyl tail controlled the distance of the interlayer space. Similar to Mt, Zeol (M*_x_*D*_y_*[Al*_x_*_+2*y*_Si*_n_*_(*x*+2*y*)_ O_2*n*_]·*m*H_2_O (D refers to the divalent exchangeable cations, such as Ca^2+^) possesses exceptional isomorphic substitution, but Zeol is a porous aluminosilicate with a homogeneous pore size, which restricts the intercalation of large molecules. According to Figure 2c, the applied Zeol is a permutite of the NaA type (PDF #11-0589, Linda 4A), which is compatible with its composition (Table 1). After magnifying the 100 reflections (Figure S1), a small shift was observed, pointing to the possibility of a structure alteration. Considering the size of the Linda 4A pore and the –N^+^(CH_3_)_3_ group (4.3 Å × 5.1 Å × 6.7 Å, [30]), the intercalation of TM or DMO seems inapplicable, which implies that the slight structural change can be ascribed to covalent grafting.

#### 3.1.2. Thermal Gravimetric Analysis

**Figure 3:**
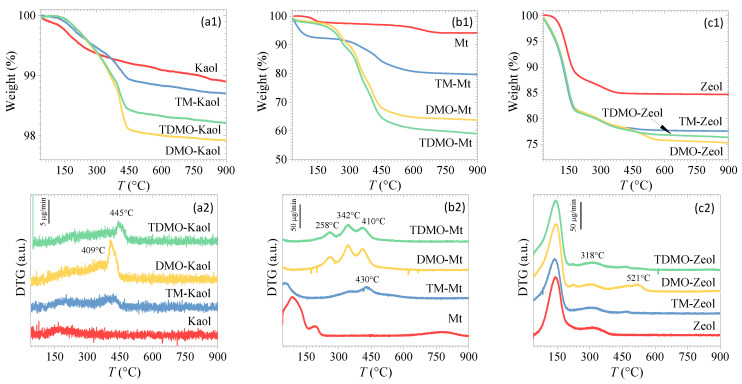
TG-DTG curves of the grafted (**a1**,**a2**) Kaol, (**b1**,**b2**) Mt, and (**c1**,**c2**) Zeol.

The TG curves were collected to estimate the content of TM and/or DMO on the grafted aluminosilicates (Figure 3). Compared with the pristine Kaol, the grafted-Kaol composites demonstrated more visible mass loss (within 1~2%, Figure 3(a1)) as a result of the successful loading of the applied organosilane. The mass losses that occurred from room temperature to 300 °C can be attributed to the physically adsorbed water, hydration water, and electrostatically and/or physically adsorbed organosilane [18]. The fact that the pristine and the grafted Kaol had a similar mass loss suggests that only a negligible amount of organosilane was loaded through electrostatic and/or physical adsorption. In contrast, significant mass losses were observed between 380 and 450 °C, resulting from the decomposition of the covalently bound organosilane [18]. In addition, the onset temperature of alkyl chain decomposition is normally higher than 200 °C in an N_2_ atmosphere [31]. Therefore, the differences in the TG curves for the three composites when the temperature ranged from 280 to 450 °C can be attributed to the different amount of methylene in TM and DMO.

When compared to Kaol, the loading of TM and/or DMO on Mt is more remarkable, as evidenced by the greater mass loss (Figure 3(b1)), which is likely due to the higher content of available hydroxyl groups on Mt. Applying DMO to graft Mt resulted in two extra peaks (~258 and 342 °C) in the DTG curves with respect to TM-Mt. The intensive steric hindrance derived from the long alkyl chain of DMO restrained the intercalation, resulting in the distribution of some DMO on the external surface. Due to the lack of protection from the Mt layers, the DMO decomposed at relatively low temperatures [32]. Interestingly, when Mt was co-grafted by TM and DMO, the amount of loaded organosilane increased. This is different from the cases of the grafted Kaol and Zeol, where the highest organosilane contents were observed when only DMO was applied. The coexisting small TM may act as a pioneer in the expandable interlayer space of Mt, increasing the interlayer distance, mitigating the steric hindrance from the Mt layers, and facilitating the intercalation of the large DMO. According to a previous study, grafting the Mt by TM and DMO in sequence may result in higher grafting efficacy [33]. Notably, the DTG curve of the alkyl quaternary ammonium-modified Mt usually show several peaks, with the more remarkable mass loss occurring at a lower temperature of about 250 °C [31,34]. This was elucidated by the different positions and binding types of the organic modifiers, i.e., external/internal distribution and hydrophobic/electrostatic interactions [35]. Three similar peaks in the DTG curves of TDMO-Mt and DMO-Mt indicate that only a relatively small amount of organosilane was deposited by the covalent grafting to the external surface. Although DMO-Mt shows a similar mass loss to a previously reported DMO-modified bentonite (~32 wt% [18]), the latter only shows two visible peaks, most likely as a result of the decomposition in a different atmosphere.

For the grafted Zeol, the mass loss assigned to the dehydration was significant (~17 wt%, Figure 3(c1)) and that attributed to organosilane decomposition ranged from 5.5% for TM-Zeol to 7.7% for DMO-Zeol. This suggests that the organosilane loading on Zeol was not greatly affected by the type of organosilane. In general, the bilayer configuration of the organosilane through the hydrophobic interactions of the long alkyl chains occurs readily [4], increasing the loading and showing a remarkable mass loss. This is contrary to the observed phenomenon (Figure 3(c1)), which showed that the same mass loss occurred for the three grafted Zeol up until ~400 °C, indicating that the loaded organosilanes were predominantly grafted via the covalent bond rather than the weak hydrophobic interactions. As a result, the release of the organosilane will be negligible, avoiding secondary pollution and ensuring their application in removing pollutants from water bodies with high requirements.

#### 3.1.3. Solid-State Nuclear Magnetic Resonance

**Figure 4:**
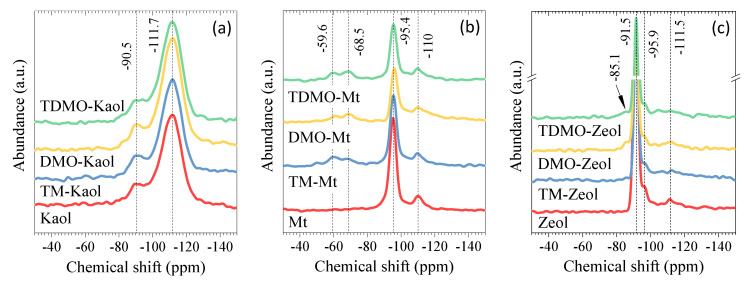
Solid−state ^29^Si−NMR spectra of the original and grafted (**a**) Kaol, (**b**) Mt, and (**c**) Zeol.

In order to investigate the grafting forms of the applied organosilanes in different composites, solid-state ^29^Si NMR spectra were collected to elucidate the Si species (Figure 4), which are conventionally notated using Q*^n^*, T*^n^*, and M*^n^*. Tetra-, tri-, and monofunctional units are referred to by the letters Q, T, and M, respectively, and n indicates the number of oxygen atoms further bonded to another silicon atom [10]. For all composites, the resonance at approximately −110 ppm, which corresponds to Q^4^ [Si(OSi)_4_], was assigned to the calcination-induced structural change (for Kaol [36]) and/or the coexisting quartz impurity. After the grafting, there was no new resonance observed because the loading of organosilane was rather small for the applied Kaol (Figure 4a). In contrast, except for the main resonance at approximately −95 ppm assigned to Q^3^ species, two new resonances appeared at −68.5 and −59.6 ppm for the grafted Mt (Figure 4b). If the condensation reaction occurred among organosilane molecules, the new resonances would be likewise observed for the case of Kaol, considering that the same amount of organosilane was used. This is inconsistent with the obtained results. In other words, the observed resonance at −68.5 and −59.6 ppm, corresponding to the T^3^ (R-Si(OSi)_3_) and T^2^ (R-Si(OSi)_2_(OMe)) species, respectively [10], solidly confirms that the condensation reaction occurred between the organosilanes and the silanol groups of Mt. Interestingly, DMO-Mt has a greater T^3^/T^2^ ratio than TM-Mt, indicating that the longer alkyl chain in the organosilane makes the full condensation reaction easier and results in more stable grafting. Similar to the case of Kaol, the grafted Zeol shows a negligible difference compared to the pristine Zeol (Figure 4c), which is partially due to the small loading of organosilane (Figure 3(c1)). However, the resonance at −85.1 ppm assigned to the Q^3^ (R-Si(OSi)_3_OH) species becomes visible after the grafting, which normally occurs with the disintegration of the structure [37]. This may be caused by the intensive synthetic conditions, including the combination of ultrasound and microwave and/or further milling for sieving after the grafting. In summary, the applied organosilanes are more readily grafted on the Mt with respect to the Kaol and Zeol.

### 3.2. Adsorption Characteristics

#### 3.2.1. Influence of Contact Time

**Figure 5:**
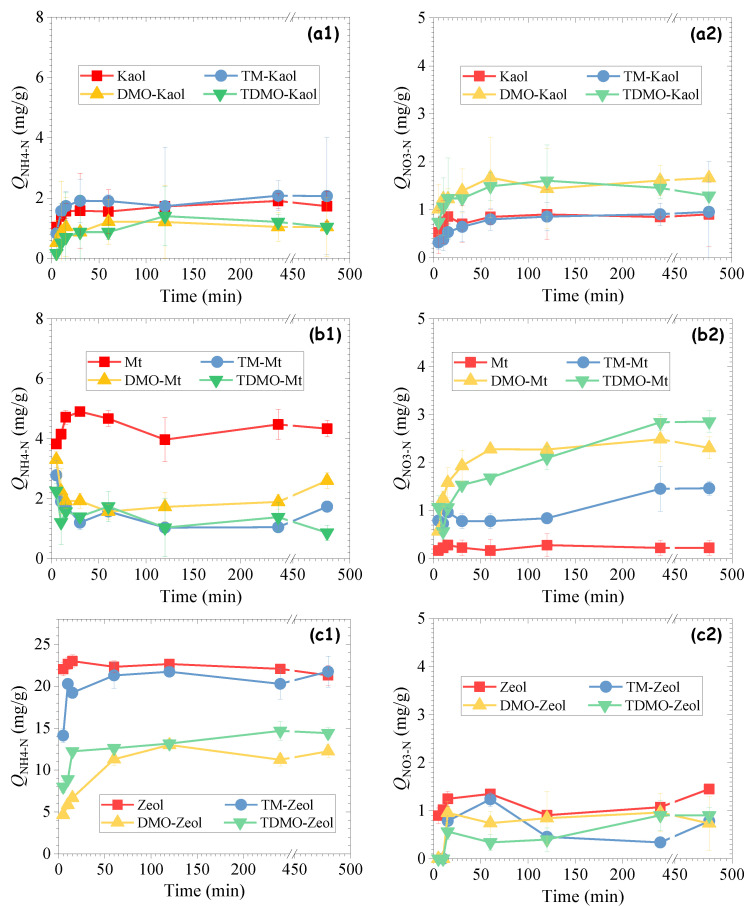
Influence of contacting time on the adsorption of NH_4_^+^-N and NO_3_^−^-N on the grafted (**a1**,**a2**) Kaol, (**b1**,**b2**) Mt, and (**c1**,**c2**) Zeol.

One of the most crucial indices for assessing the performance of an adsorbent is the adsorption rate [38]. As shown in Figure 5, the grafting decreased the NH_4_^+^ uptake without any significant influence on the adsorption rate. It is well-known that NH_4_^+^ is mainly adsorbed on aluminosilicate minerals via cation exchange. However, because the grafting introduced positively charged -R_4_N^+^ groups and reversed the surface charge from negative to positive, NH_4_^+^ was repulsed to proceed with the ion exchange process. Notably, the adsorption mitigation became more significant when the long alkyl chain-bearing DMO was used, which can be ascribed to the higher loading of organosilane (Figure 3). In the case of Kaol, the amount of adsorbed NH_4_^+^-N equilibrated at 1~2 mg/g within 30 min, which is comparable with the grafted Mt and significantly lower than the grafted Zeol. This suggests that the active adsorption sites for NH_4_^+^ were rapidly occupied within 30 min [38], and the adsorbed NH_4_^+^ repelled the free NH_4_^+^ in solution to achieve a balance with the concentration gradient-derived driving force after 30 min [39]. Different from NH_4_^+^ uptake, the adsorption of NO_3_^−^ on the grafted aluminosilicate minerals should be mainly attributed to the anion exchange with the chloride ion that neutralizes the -R_4_N^+^ group. However, although the grafted Zeol samples show moderate amounts of organosilane, the adsorption of NO_3_^−^ on them was less favorable than on the grafted Kaol or Mt. This might be caused by the neutralization of the -R_4_N^+^ group of the covalently bonded organosilane by the negatively charged Zeol. As evidenced by the highest concentration of organosilane in the grafted Mt (Figure 3), a greater number of available -R_4_N^+^ groups were involved, accounting for the best performance in regard to NO_3_^−^ uptake. Notably, although the parallel procedure was applied for all experiment test, the visible error bars for some points still existed, which is believed to be caused by the heterogeneity of the composites, such as the existence of quartz impurity (Figure 4).

#### 3.2.2. Influence of the Initial Concentration

**Figure 6:**
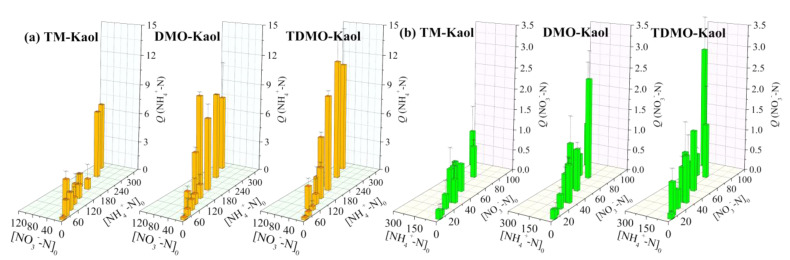
Influence of initial concentration on the adsorption of (**a**) NH_4_^+^−N and (**b**) NO_3_^−^−N using the grafted Kaol.

To understand the influence of the initial concentration of NH_4_^+^ or NO_3_^−^ on the adsorption performance of the developed composites, a batch of solutions with different concentrations of NH_4_^+^-N and NO_3_^−^-N was prepared (Table S1). The increase in the initial concentration increased the force against the resistance of mass transfer between adsorbent and adsorbate, accounting for the increased uptake of pollutants [38]. As shown in Figure 6, the grafted Kaol by TM show the lowest uptake of NH_4_^+^-N and NO_3_^−^-N with respect to the other two. Overall, the amount of adsorbed NH_4_^+^-N or NO_3_^−^-N increased with the individual initial concentration, suggesting that the adsorption of NH_4_^+^ and NO_3_^−^ on the grafted Kaol was non-interfering. The adsorption characteristics of NH_4_^+^ or NO_3_^−^ on DMO-Kaol and TDMO-Kaol were similar, indirectly implying that the grafting of DMO rather than TM governed the adsorption performance. The amount of adsorbed NH_4_^+^ on TDMO-Mt was around 10 mg/g, which is lower than a metakaolin-derived geopolymer [40] but comparable to a thermoactivated kaolinite (*C*_0_ = 200 mg/L) [41]. For the case of NO_3_^−^, the observed uptakes of most tested systems fluctuate at 1.0 mg/g, which is smaller than the adsorption capacity of positive charge-induced kaolinite [42]. This might be attributed to the differences in the starting kaolinite and the modification protocol. Notably, the impact of increasing the initial concentration on the adsorption quantity is more significant for NH_4_^+^ than for NO_3_^−^, likely due to the different adsorption mechanism (i.e., cation exchange and anion exchange, respectively).

**Figure 7:**
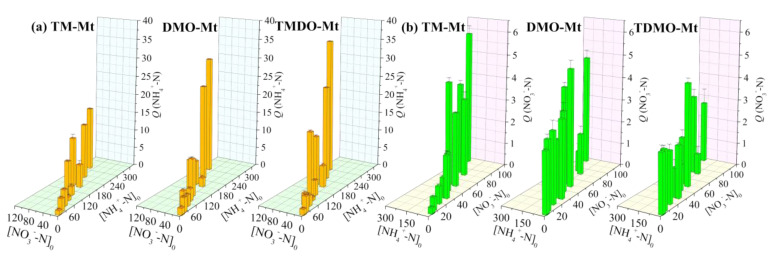
Influence of initial concentration on the adsorption of (**a**) NH_4_^+^−N and (**b**) NO_3_^−^−N using the grafted Mt.

Due to the high cation exchange capacity (CEC) of Mt (0.911 meq/g), the amount of adsorbed NH_4_^+^ or NO_3_^−^ on the grafted Mt was significantly higher than that on the grafted Kaol (Figure 7). Although NH_4_^+^ uptake increased visibly with [NH_4_^+^-N]_0_, [NO_3_^−^-N]_0_ also played an important role in the uptake increase. With a higher concentration of NO_3_^−^, the more positive charges of the grafted Mt are neutralized and/or screened, mitigating the repulsive force to NH_4_^+^ and allowing the penetration of NH_4_^+^ into the interlayer space to reach the adsorption sites. At the high concentrations of [NH_4_^+^-N]_0_ and [NO_3_^−^-N]_0_, the uptake of NH_4_^+^-N exceeded the CEC of Mt, which might be caused by formation of ammonium salts in the interlayer space [43]. In contrast, for NO_3_^−^ uptake, the influence of [NO_3_^−^-N]_0_ varied with the type of grafted Mt. For DMO-Mt and TDMO-Mt, the amount of adsorbed NO_3_^−^ was considerable even at low [NO_3_^−^-N]_0_, suggesting the high affinity to NO_3_^−^. The high selectivity between alkyl quaternary ammonium-functionalized Mt and NO_3_^−^ has also been reported [16]. Moreover, in contrast to the case of NH_4_^+^ uptake, the adsorption of NO_3_^−^ seems to be negligibly influenced by [NH_4_^+^-N]_0_. Given that a certain amount of organosilane was electrostatically loaded in the composites, the desorption of the organosilane may occur in the solution with high [NH_4_^+^-N]_0_ [9], releasing the Mt-neutralized -R_4_N^+^ groups, providing new adsorption sites for NO_3_^−^, and increasing the uptake. However, this explanation is inconsistent with the observed phenomenon. This implies that there was only a negligible amount of organosilane that was electrostatically immobilized or the loaded organosilane was hard to desorb in the grafted Mt, showing favorable stability and coinciding with the insignificant release of organosilane [18]. This is also supported by the negligible amount of bubbles observed in the adsorption vial after vigorous shaking.

**Figure 8:**
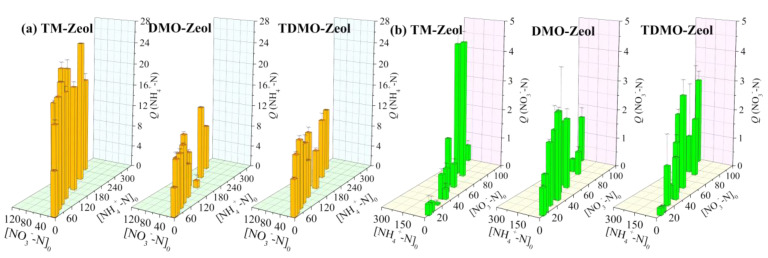
Influence of initial concentration on the adsorption of (**a**) NH_4_^+^−N and (**b**) NO_3_^−^−N using grafted Zeol.

Among the three aluminosilicates, TM-Zeol showed the best performance in terms of NH_4_^+^ uptake, which was around 18 mg/g in all the examined systems (Figure 8). However, the use of DMO reduced the ability to adsorb NH_4_^+^, with an average uptake of ~8 mg/g for DMO-Mt and TDMO-Mt, which is comparable to the previously reported natural zeolite [44]. This could be ascribed to the coverage or blockage of the penetration pathway of NH_4_^+^ into the interior adsorption sites [45]. As opposed to the grafted Kaol and Mt, the adsorption of NH_4_^+^-N on the grafted Zeol was negligibly impacted by the [NH_4_^+^-N]_0_ and [NO_3_^−^-N]_0_, suggesting high affinity and non-interference, respectively. In most of the investigated systems, the amount of adsorbed NO_3_^−^ on the grafted Zeol were less than 2.0 mg/g. In general, this is superior to that on the grafted Kaol but inferior to that on the grafted Mt, which is in good agreement with the organosilane content. In other words, the organosilane content in the grafted aluminosilicate determines how much NO_3_^−^ can be adsorbed.

## 4. Conclusions

Three aluminosilicate minerals, including calcined kaolinite (Kaol), montmorillonite (Mt), and zeolite (Zeol), were modified by trimethyl [3-(trimethoxysilyl) propyl] ammonium chloride (TM) and/or dimethyl octadecyl [3-(trimethoxysilyl) propyl] ammonium chloride (DMO), and the resulting composites were used to remove and/or recover NH_4_^+^ and NO_3_^−^ from an aqueous solution to eliminate environmental hazards. Grafting of TM and/or DMO exhibited a negligible influence on the crystal structures of Kaol and Zeol, but expanded the interlayer space of Mt compared to Kaol and Zeol. Mt held more organosilane and showed the greatest loading concentration when TM and DMO were used simultaneously. According to the ^29^Si-NMR spectra, TM and/or DMO were covalently bonded on Mt, while this was difficult to identify for Kaol and Zeol due to the low concentration of organosilane. The grafting of TM and/or DMO decreased the uptake of NH_4_^+^ without any significant influence on the adsorption rate. The adsorption of NH_4_^+^ and NO_3_^−^ on the grafted Kaol and Zeol was non-interfering, while the coexisting NO_3_^−^ facilitated NH_4_^+^ uptake on the grafted Mt. The loading of DMO improved the affinity to NO_3_^−^ and its concentration determined the adsorption amount. The findings suggested that the grafted Mt may be able to simultaneously decontaminate the cationic and anionic pollutants from environmental media, such as NH_4_^+^ and NO_3_^−^ in the wastewater of ionic rare earth mining, Cs^+^ and TcO_4_^−^ in radioactive wastewater, etc. However, considering the separability, adsorption performance, and cost of the organosilane, hybridization with other highly separable materials (e.g., Fe_3_O_4_, polymers) and the deployment of other cost-effective R_4_N^+^-bearing modifiers will further facilitate the application of the developed materials in actual wastewater treatment.

## Figures and Tables

**Table 1:** Concentrations (%) of main compositions in the used Kaol, Mt, and Zeol.

Sample	SiO_2_	Al_2_O_3_	MgO	CaO	Fe_2_O_3_	Na_2_O	K_2_O	TiO_2_
Kaol	44.88	44.03	0.11	0.25	0.41	0.21	0.13	1.39
Mt	67.06	16.26	3.80	2.47	1.94	0.23	0.55	/
Zeol	34.36	28.50	0.28	0.15	0.01	17.67	0.07	0.01

## Data Availability

All data generated or analyzed during this study are included in this article.

## Supplementary Materials

The following supporting information can be downloaded at: https://www.mdpi.com/article/10.3390/ijerph191912562/s1, Table S1: Initial concentrations of NH_4_^+^-N and NO_3_^−^-N in different adsorption systems; Figure S1: Enlarged XRD patterns for (100) reflections of the original and grafted Zeol.
