# Supplementary material for: Grafting of R4N+-Bearing Organosilane on Kaolinite, Montmorillonite, and Zeolite for Simultaneous Adsorption of Ammonium and Nitrate
Source: Int J Environ Res Public Health. 2022 Oct 1;19(19):12562. doi: 10.3390/ijerph191912562 (PMC9566248; doi:10.3390/ijerph191912562)
Supplement: Supplementary file 1 [file ijerph-19-12562-s001.zip › ijerph-1954165-supplementary.pdf]

Table S1. Concentrations of NH_4^+–N and NO_3^-–N in different adsorption systems.

Adsorption systems	Adsorbate concentration (mg/L)	
	$[NH_4^+\text{-N}]_0$	$[NO_3^-\text{-N}]_0$
1	10	5
2	25	10
3	50	20
4	50	30
5	75	30
6	100	40
7	100	50
8	150	50
9	150	75
10	200	75
11	250	100

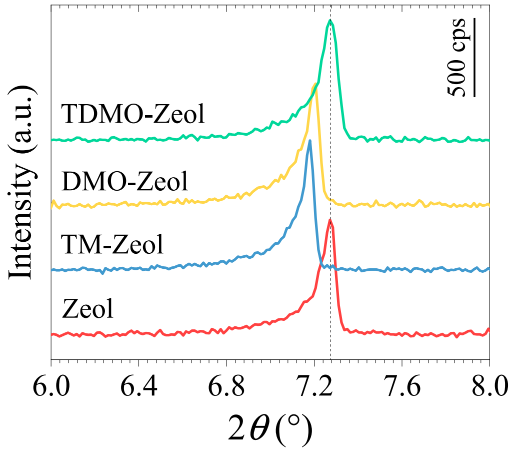

Figure S1. Enlarged XRD patterns for (100) reflections of the original and grafted Zeol.
